# Changes in safety climate and teamwork in the operating room after implementation of a revised WHO checklist: a prospective interventional study

**DOI:** 10.1186/s13037-017-0120-6

**Published:** 2017-01-31

**Authors:** Sofia Erestam, Eva Haglind, David Bock, Annette Erichsen Andersson, Eva Angenete

**Affiliations:** 1 Department of Surgery, Institute of Clinical Sciences, Sahlgrenska Academy at University of Gothenburg, Gothenburg, Sweden; 2 SSORG - Scandinavian Surgical Outcomes Research Group, Sahlgrenska University Hospital, SE-416 85 Gothenburg, Sweden; 3 Institute of Health and Care science, University of Gothenburg, Gothenburg, Sweden

**Keywords:** Patient safety, Operating room, Safety climate, Teamwork, WHO checklist

## Abstract

**Background:**

Inter-professional teamwork in the operating room is important for patient safety. The World Health Organization (WHO) checklist was introduced to improve intraoperative teamwork. The aim of this study was to evaluate the safety climate in a Swedish operating room setting before and after an intervention, using a revised version of the WHO checklist to improve teamwork.

**Methods:**

This study is a single center prospective interventional study. Participants were personnel working in operating room teams including surgeons, anesthesiologists, scrub nurses, nurse anaesthetists and nurse assistants. The study started with pre-interventional observations of the WHO checklist use followed by education on safety climate, the WHO checklist, and non-technical skills in the operating room. Thereafter a revised version of the WHO checklist was introduced. Post-interventional observations regarding the performance of the WHO checklist were carried out. The Safety Attitude Questionnaire was used to assess safety climate at baseline and post-intervention.

**Results:**

At baseline we discovered a need for improved teamwork and communication. The participants considered teamwork to be important for patient safety, but had different perceptions of good teamwork between professions. The intervention, a revised version of the WHO checklist, did not affect teamwork climate. Adherence to the revision of the checklist was insufficient, dominated by a lack of structure.

**Conclusions:**

There was no significant change in teamwork climate by use of the revised WHO checklist, which may be due to insufficient implementation, as a lack of adherence to the WHO checklist was detected. We found deficiencies in teamwork and communication. Further studies exploring how to improve safety climate are needed.

**Trial registration:**

NCT02329691.

## Background

Each year approximately 234 million surgeries are performed worldwide, and in 3 – 16% patients suffer from major complications [1]. To reduce complications and improve results after surgery both technical and non-technical skills are required [2]. The operating room team consists of many professions, which complicates the teamwork. Collaboration between team members from different disciplines and with different educations requires comprehensive coordination and cooperation. Basic structure and mutual respect as well as team structure and a shared mental model allow individual team members to understand and appreciate their own role as well as those of others, resulting in more effective communication [3, 4]. It is important that the basic structure is well-known by all team members both outside and inside the operating room. Stout, et al. [3] describes a shared mental model to provide the team members with a shared understanding of the team task and knowledge about who is responsible for what. This allows the team to anticipate one another’s needs so that they can work as an effective team and make successful decisions.

In 2007 the World Health Organization (WHO) study group ‘Safe surgery saves lives’ created a checklist: the WHO Surgical Safety Checklist with the purpose to improve intraoperative team communication and consistency of care. Implementation of the checklist was found to reduce postoperative morbidity and mortality [5]. Insufficient use of and/or missing items in the WHO checklist may provide a false sense of security for the operating team [6].

Two concepts, safety culture and safety climate are common when discussing safer surgery. Safety culture has been described as reflections on the fundamental values of an organization as well as norms, assumptions and expectations. Safety climate [7] entails the employee’s perceptions, awareness, beliefs and attitudes about risk and safety and has been measured using questionnaires such as the Safety Attitudes Questionnaire (SAQ) [8].

There is a lack of knowledge regarding the safety climate in Swedish operating room settings [9]. The aim of this study was to evaluate the teamwork and the safety climate in a Swedish operating room setting before and after implementation of a revised version of the WHO checklist. Our hypothesis was that by using the WHO checklist in a structured fashion and by adding a description of the surgical procedure and patient, we would increase commitment and enhance the teamwork.

## Methods

### Setting and participants

**Fig. 1:**
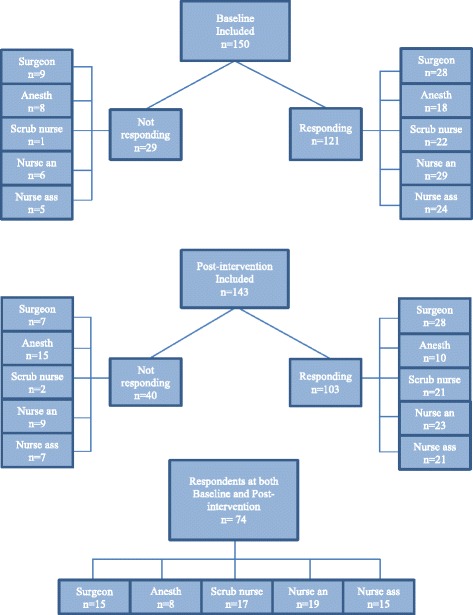
Flow chart for participants in the study

The study was conducted at Sahlgrenska University Hospital, Gothenburg, Sweden. Participants were personnel working in operating room teams. The teams consisted of the following professional groups: surgeons, anesthesiologists, scrub nurses, nurse anaesthetists and nurse assistants. Together these professional groups consisted of 150 personnel (Fig. 1). Two collaborating organizational units within the hospital were involved in this study, the Department of Surgery and the Department of Anaesthesia. All but the surgeons were formally employed by the Department of Anaesthesia. Depending on the surgical procedure the number of team members present in the operating room differs, but in most cases the team consists of one anesthesiologist, one nurse anaesthetist, two-three surgeons, one scrub nurse and one nurse assistant. In each operating room approximately 2–5 procedures are performed daily. The anesthesiologists were responsible for several simultaneously ongoing surgical procedures, and were seldom present in the operating room for the review of the WHO checklist. The nurse anaesthetists were present in the operating room throughout the procedure. The nurse assistants assist both the scrubbed and the anaesthetic team.

### Study design

**Fig. 2:**
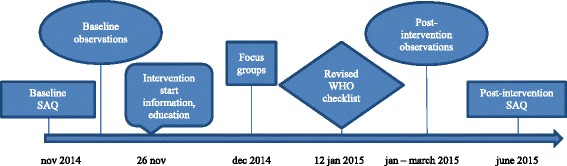
Timeline for the study

This is a single center prospective interventional study. Chronological order for the study is demonstrated in Fig. 2. The study period lasted 7 months, from November 2014 until June 2015. The study started with the questionnaire SAQ measuring baseline (Nov 2014) followed by baseline observations of the use of the original WHO checklist (Nov 2014). The intervention period started with information and education (Nov 26 2014) followed by Focus groups (Dec 2014) and implementation of the revised WHO checklist (Jan 12 2015). Post-intervention observations of the revised checklist were performed (Jan-March 2015) and the final SAQ post-intervention was measured (June 2015). Prior to study start the operating room management consented to the implementation and the study.

### Baseline measurements

#### Baseline WHO checklist

The Swedish version of the checklist was produced by LÖF in 2009 [10]. In the operating rooms we studied the WHO checklist had been in daily use since 2009, but without previous evaluation. The implementation of the checklist in 2009 consisted of a meeting with information, including a film sequence about the importance of the WHO checklist. The nurse assistant was assigned the role as checklist coordinator. Shortly after introduction a customized revision of the checklist was made to tailor it to the needs of this operating ward. A laminated copy was available in each operating room.

#### Baseline Safety Attitudes Questionnaire (SAQ)

The intervention was evaluated with the SAQ - operating room (OR) version. The version used in this study is derived both from the original SAQ OR version and from a translated, validated Swedish version [9, 11]. Two items not previously translated were used, the first was ‘Use the scale to describe the quality of communication and collaboration you have experienced with: surgeons, anesthesiologists, scrub nurses, nurse anaesthetists and nurse assistants’ The second was the open ended question ‘What are your top three recommendations for improving patient safety in the operating room?’ These two items were back-and-forward translated and face-to-face validated, before use. SAQ contains six domains: teamwork climate, safety climate, perception of management, job satisfaction, working conditions and stress recognition.

The items in SAQ are on a 5 point Likert type-scale, anchored by 1 = disagree strongly and 5 = agree strongly. Two of the items, 12 and 24 had a reversed anchoring and were re-coded prior to analysis. Individual items are reported as above while scores were calculated for each domain. The domain scales were transformed into a score scaled 0–100 [7, 12]. The collaboration and communication items of SAQ, anchored by 1 = very low and 5 = very high, were dichotomized with the cut off >3 (adequate).

SAQ was distributed two months prior to (baseline) and two weeks after the end of the intervention (post-intervention), respectively. SAQ was handed out during staff meetings and personnel not attending such meetings received it through the hospital’s internal mail. Each questionnaire contained a unique study ID and study information and pre-addressed return envelopes were attached. After two weeks a reminder was posted.

#### Baseline structured observations

Prior to the intervention observations were made at baseline to evaluate the use of the original WHO checklist. This was done by using a pre-defined Clinical Record Form (CRF). The CRF consisted of both structured questions and field notes in the form of descriptive and reflective notes. Observations continued until saturation, when the data set was complete and nothing new was being added. Saturation ensures that data is comprehensive and complete [13]. One of the authors (SE) performed all observations. The observer briefly explained her presence in the operating room before the start of the procedure, and did not comment on how the checklist was used.

### The intervention

A key component of how the intervention was designed was focus group meetings with the participants, aiming at using ideas and experiences of the staff to adapt and improve the original WHO checklist. This was followed by educational sessions and dialogue meetings with participants and finally the implementation of the revised WHO checklist.

#### Focus groups

The personnel participating in the focus groups were divided by professional categories into six focus groups (surgeons divided into 3 groups dependent on surgical specialty, scrub nurses, nurse anesthetists, nurse assistants) and the focus groups were led by one of the authors (SE) [14]. The focus groups consisted of 10–20 participants at each occasion. The anesthesiologists did not recognize the need for further education and did not participate in this part of the intervention. The focus groups started with information about the WHO checklist and possible improvements of the checklist were discussed. The idea of adding the item ‘description of the surgical procedure’ to the WHO checklist was presented to the participants. Three open-ended questions were asked: ‘How can we work with the WHO checklist to improve patient safety?’, ‘What parts are well functioning today?’, ‘Are there any parts of the WHO checklist that need revision?’ Information from the focus groups was used to construct a revised version of the WHO checklist.

Data from focus groups were analyzed using a qualitative content analysis [15]. The focus group dialogues were recorded and then transcribed. The texts were initially read multiple times to identify the main focus. The text was divided into meaning units that were condensed and categorized [15]. The interpretations were done by two of the authors (SE, AEA).

The qualitative content analysis of the six focus groups resulted in two categories described below [15].

#### Inadequate structure concerning the WHO checklist

There was uncertainty regarding who was the designated checklist coordinator and this was described as confusing and causing lack of focus. The nurse assistants found it difficult to initiate ‘Time out’ as their role was insufficiently recognized. They also felt that the surgeons had a lack of focus and gave the last part of the checklist, ‘Sign out’ a low priority and this was confirmed by the surgeons themselves. The nurse assistants were in charge of the hospital phones in the operating room, but they were uncertain about how to handle incoming calls for the surgeons, who have to be reachable when they are responsible for a surgical ward. Many surgeons also left their private mobile phone with the nurse assistants and as the surgeons’ preferences differed, the ‘phone question’ was a problem. Surgeons expressed that frequent changes of team members during a procedure required repeated ‘Time out’ for the WHO checklist to remain meaningful. The nurse anaesthetists suggested that ‘Sign out’ should be completed during wound closure before it was possible for the surgeons to leave the operating room. Information from the last item ‘What can we learn, what can we do better next time?’ was suggested to be saved for future improvements.

#### Benefits of improved description of the surgical procedure

*‘It is really great that you have increased the focus on the patient, we should all have that focus.’* All groups responded positively to a revision of the checklist with a more detailed description of the surgical procedure. The surgeons saw the description of the surgical procedure as an opportunity to educate the team on what was important for the specific operation.

#### Educational settings

All participants were invited to informative and educational events, including inter-professional lectures in large groups. On these occasions the topics safety culture, safety climate in health care, the importance of non-technical skills in the operating rooms and the importance of WHO checklist were covered. Information was also sent to the participants by e-mail on several occasions.

#### The revised WHO checklist

In the revised WHO checklist four changes were made to checklist procedure:

1. The checklist was filled out on paper for each surgical procedure, and the checklist coordinator checked each item box with a pen to ensure that all items were reviewed.
2. For ‘Sign in’, one question was added: ‘Presence of metal implant?’ to remind the nurse anaesthetist to ask the patient, and to report the answer to the team.
3. At ‘Time out’ a section called ‘Description of the surgical procedure’ was added. It included a more thorough explanation of the underlying indication for surgery and, information about the surgical procedure and the patient. The intention was to increase the clinical understanding in the team and thereby improve the shared situational awareness and the team work.
4. At ‘Time out’ ‘How to manage incoming telephone calls?’ was added as a help to the nurse assistant to address incoming calls to the surgeon during surgery, according the surgeon’s own preference.

Before the implementation of the revised checklist participants were once again gathered in groups. The entire staff was informed about the changes to the checklist through information on meetings, e-mails and informative memos.

### Post-intervention

#### Structured observations during use of the revised WHO checklist

Structured onsite observations was one of the evaluation tools used to evaluate the use of the revised version of the WHO checklist. The revised checklist was implemented on 12 January. During the period, 12 January to 12 May 2014, 1267 checklists were used, whereof 264 (21%) were completely filled out, with no omissions. Thirty-five structured observations were conducted during this period. The observational data were analyzed and categorized in relation to: ‘Sign in’, ‘Time out’ and ‘Sign out’.

#### Post-intervention Safety Attitude Questionnaire (SAQ)

SAQ was used both at baseline and post-intervention. In order to assess the ‘teamwork climate’, communication and collaboration among different professions was analyzed.

SAQ post-intervention was distributed two weeks after the end of the period of using the revised WHO checklist. SAQ was once again handed out during staff meetings and personnel not attending the meetings received the questionnaire through the hospital’s internal mail.

### Analysis methods

#### Qualitative analysis

Focus groups, observations, and the open-ended question from SAQ ‘What are your top three recommendations for improving patient safety in the operating room?’ were analyzed using a qualitative content analyzes [15]. The observations and the SAQ were divided into time-sequences before abstraction. The analysis was conducted using NVivo 10, qualitative data analysis Software (QSR International Pty Ltd. Version 10, 2012).

#### Statistical analysis

For domain scores, intra-individual changes as well as between professional categories were evaluated by paired *t*-test and analysis of covariance, respectively. Software used were SPSS, version 22 (SPSS).

## Results

### Baseline Safety Attitudes Questionnaire (SAQ)

The operating rooms studied had a staff of 150 persons, including surgeons, anesthesiologists, scrub nurses, nurse anaesthetists and nurse assistants (Fig. 2), of whom 121 (81%) answered the baseline questionnaire.

‘Job satisfaction’ at baseline showed the highest score (Table 1). The lowest score was found in ‘perception of management’.

**Table 1:** SAQ domain scores at baseline and post-intervention

Domain^a^	Baseline (n)	Baseline	Post-inter-vention (n)	Post-intervention	(n) ^b^	Mean Change	SEM’	*P*-value
		Mean (SD)		Mean (SD)				
Teamwork climate	106	65 (15.2)	88	64 (13.2)	63	0.3	1.4	ns
Job satisfaction	114	75 (15.2)	94	72 (14.2)	65	−2.2	1.3	ns
Perception of management	106	58 (16.3)	86	53 (16.1)	59	−5.4	2.2	0.016
Safety climate	97	62 (14.9)	81	61 (13.9)	52	1.8	2.0	ns
Stress recognition	118	70 (19.6)	93	72 (17.7)	66	1.9	2.0	ns
Working conditions	95	64 (15.0)	74	61 (14.4)	47	−1.2	1.8	ns

Domain Score in SAQ before (baseline) and after (post-intervention) the use of the revised version of the WHO checklist

Standard Error of the Mean change

^a^ All domains on a scale 0–100, presented as mean value where 0 = Disagree strongly, 100 = Agree Strongly. Scores over 75 are taken as positive

^b^ Participants answering both SAQ baseline and SAQ post-intervention

At baseline, surgeons and anesthesiologists scored significantly higher than nurses regarding ‘Teamwork climate’ (72.2 SD 10 vs 62.2 SD 16.2 *p* = 0.001) for details see Table 2.

**Table 2:** SAQ domain scores, a comparison between doctors and nurses

Domain	Profession	Baseline				Post-intervention				Change			
		(n)	Mean	(SD)	*p*-value	(n)	Mean	(SD)	*p*-value	(n) ^a^	Mean	SEM	*P*-value
Team work climate	Doctors	34	72	10.0	0.001	27	69	11.2	0.006	16	−1.8	1.6	0.700
	Nurses	72	62	16.2		61	61	13.2		47	1.0	2.8	
Job satisfaction	Doctors	42	75	14.6	0.856	37	73	12.3	0.551	22	−1.2	2.1	0.498
	Nurses	71	75	15.7		57	71	15.3		43	−2.7	1.7	
Perception of management	Doctors	37	63	17.9	0.023	31	57	16.3	0.081	18	1.4	2.2	0.005
	Nurses	69	56	14.9		55	51	15.7		41	−8.4	2.9	
Safety Climate	Doctors	30	66	14.9	0.056	30	63	14.0	0.307	14	−1.8	3.4	0.907
	Nurses	67	60	14.6		51	60	13.9		38	3.1	2.4	
Stress recognition	Doctors	44	76	16.8	0.013	36	73	14.0	0.690	23	−2.2	3.3	0.638
	Nurses	73	66	20.6		57	71	19.8		43	4.1	2.5	
Working Conditions	Doctors	30	70	15.2	0.005	26	65	15.6	0.072	11	2.3	4.4	0.132
	Nurses	65	61	14.1		48	59	13.4		36	−2.3	2.0	

Domain Score in SAQ at two different time points reported in the different domains, before (baseline) and after (post-intervention) a change in the use of WHO checklist. All domains on a scale 0–100, presented as mean value where 0 = Disagree strongly, 100 = Agree Strongly. *P*-values are a comparison between doctors and nurses perception of different domains. Doctors = surgeons and anesthesiologist. Nurses = scrub nurses, nurse anaesthetists, nurse assistants

^a^Participants answering both SAQ baseline and SAQ post-intervention

The analysis of separate items in ‘Teamwork climate’ showed that doctors appreciated input from nurses while nurses did not perceive this (Table 3). The same pattern was found in the item ‘I have the support I need from other personnel to care for our patients’. The anesthesiologists experienced that doctors and nurses work as a well-coordinated team, while the other members of the team did not.

**Table 3:** Teamwork climate items

Teamwork Climate Items	Overall Baseline	Post-intervention	Surgeons Baseline	Post- intervention	Anesth^a^ Baseline	Post- intervention	Scrub nurses Baseline	Post-intervention	Nurse an^b^ Baseline	Post-intervention	Nurse ass^c^ Baseline	Post-intervention
Nurse input is well received in this clinical area	3.75 (0.8); 66	3.75 (0.82); 66	4.04 (0.56); 71	3.95 (0.79); 61	4.31 (0.7); 78	4.4 (0.7); 90	3.41 (0.85); 50	3.47 (0.96); 55	3.57 (0.74); 64	3.58 (0.78); 71	3.63 (0.82); 67	3.67 (0.66); 67
Disagreements in this clinical area are appropriately resolved	2.81 (0.96); 26	2.88 (0.84); 21	3.41 (0.8); 39	3.38 (0.59); 32	3.31 (0.7); 39	3.5 (0.53); 50	2.27 (0.83); 9	2.16 (0.76); 5	2.59 (0.8); 10	2.75 (0.74); 16	2.67 (1.13); 33	2.89 (0.83); 14
I have the support I need from other personnel to care for our patients	4.07 (0.74); 85	3.97 (0.73); 82	4.29 (0.46); 100	3.89 (0.83); 82	4.12 (0.86); 78	4.1 (0.57); 90	3.73 (0,7); 68	3.6 (0.82); 60	4.11 (0.69); 90	4.21 (0.51); 96	4.04 (0.95); 83	4.11 (0.68); 81
It is easy for personnel in this clinical area to ask questions when there is something that they do not understand	3.99 (0.79); 81	3.79 (0.79); 65	4.11 (0.51); 89	3.92 (0.74); 71	4.18 (0.53); 89	4.2 (0.42); 100	3.68 (0.89); 63	3.75 (0.85); 60	3.79 (0.88); 75	3.42 (0.78); 50	4.25 (0.9); 88	3.89 (0.83); 62
The doctors and nurses here work together as a well-coordinated team	3.62 (0.86); 68	3.58 (0.86); 66	3.42 (0.9); 50	3.41 (0.93); 54	4 (0.71); 83	4 (0.47); 90	3.27 (0.89); 59	3.35 (1.04); 60	3.68 (0.67); 71	3.71 (0.69); 75	3.83 (0.87); 83	3.67 (0.84); 67
In this clinical area it is difficult to speak up if I perceive a problem with patient care (reversedscored)	3.5 (1.03); 57	3.49 (0.93); 57	3.63 (1.01); 61	3.92 (0.8); 75	3.83 (0.92); 78	3.7 (0.48); 70	3.05 (0.95); 32	3.26 (0.99); 45	3.61 (0.96); 68	3.38 (1.01); 63	3.39 (1.2); 46	3.2 (0.95); 33

Data presented on a scale 0 – 5, where 0 = Disagree strongly, 5 = Agree Strongly. Presented as mean, (SD), percent agreement of positive responses. The last question is negatively worded and the score has been reversed

^a^ Anesth = Anesthesiologists

^b^ Nurse an = Nurse anaesthetists

^c^ Nurse ass = Nurse assistants

At baseline there were discrepancies between perceptions of good communication between professional groups. Most professions found the communication within their group to be the best (Table 4).

**Table 4:** Quality of communication and collaboration between operating room team members

Profession	Surgeons		Anesthesiologists		Scrub nurses	Nurse anaesthetists	Nurse assistants
	Consultants Attendings	Residents Interns	Consultants Attendings	Residents Interns			
Surgeons (*n* = 28)	*93*	*64*	50	50	64	46	36
Anesthesiologists (*n* = 18)	33	33	*56*	*67*	56	83	61
Scrub nurses (*n* = 22)	36	27	23	18	*77*	77	59
Nurse anaesthetists (*n* = 28)	29	14	75	71	68	*82*	50
Nurse assistants (*n* =24)	42	17	46	42	75	71	*71*

Communication and collaboration as appreciated among professions at baseline. Presented as percentage of participants who answered “high” or “very high”. *Italics* when a profession estimated communication and collaboration within their own profession

### Baseline structured observations

The observations revealed that checklist items were often omitted. At ‘Time out’ the most commonly omitted items were ‘What are the critical or unexpected steps?’, ‘Expected operative duration?’, and ‘Anticipated blood loss?’ whereas ‘Specimen labelling’ and ‘What can we learn from this procedure, what can we do better next time?’ were the most commonly omitted items at ‘Sign out’.

### Structured observations during use of the revised WHO checklist

#### Sign in

##### Deficiency in coordination and structure regarding the performance of ‘Sign in’

‘Sign in’ was mostly performed after ‘Time out’. In most cases the nurse assistant handed the checklist to the nurse anaesthetists for completion without involvement of other team members’ and after ‘Time out’.

#### Time out

##### The quality of the performance varied depending on individual team members

Focus from all team members was essential in order to perform ‘Time out’ adequately. On occasion the nurse assistant was ignored, or team members didn’t communicate or listen, or answered just some of the items. On other occasions the nurse assistant read only part of the checklist. The ‘description of the surgical procedure’ was often incomplete, to the dissatisfaction of the nurses. Some of the surgeons seemed reluctant to perform this part of the checklist.

#### Sign out

##### Lack of structure and clear guidelines reduced focus

At ‘Sign out’ the team often appeared unfocused. On most occasions ‘Sign out’ was conducted in an unstructured fashion with a lack of leadership.

### Post-intervention Safety Attitude Questionnaire (SAQ)

103 (72%) participants answered the SAQ post-intervention, but only 72 of the participants responded at both Baseline and Post-intervention (Fig. 2). Among these 72 there was no change in average domain scores associated with the use of the revised checklist apart from the domain ‘Perception of management’ which decreased significantly (Table 1). There was no change in teamwork climate or communication and collaboration between professions from baseline to post-intervention. Among the different professions, the nurse assistants reported an improvement in safety climate. Physicians scored significantly higher than nurses (69 SD 11.2 vs 61 SD 13.2 *p* = 0.006), in the domain ‘Teamwork climate’, both before and after the intervention, for details see Table 2.

### Safety attitude questionnaire — open ended question

Eighty-seven (73%) participants answered the open-ended question at baseline and 68 (67%) in the post-intervention questionnaire rendering 438 suggestions on how to improve patient safety.

When analyzed the answers from baseline and post-intervention did not differ and together the answers resulted in two categories.

#### Knowledge and mastering of non-technical skills to improve patient safety

The most comprehensive category contains various aspects of the operating room teams’ perceptions of non-technical skills and their importance for patient safety in the operating room. Fifty participants representing all professional categories mentioned the WHO checklist as an improvement of patient safety. Many commented on the significance of the team focus during the review of the checklist, and on the importance of always using the checklist. At baseline, there was a demand for more extended information about the surgical procedure during ‘Time out’. Surgeons, anesthesiologists and nurses listed pre-operative planning such as detailing needs for specific instruments, patient position as well as anatomical steps during the surgical procedure as important for patient safety. Improving the **c**ommunication within the operating room was considered very important by all professions. Many of the participants commented on the importance of an open climate where everyone is free to speak up and communicate with each other independent of status and profession. Eighty-eight comments from all professions were made regarding the importance of enhanced teamwork to increase the dedication from the team in the operating room. Cooperation, kindness and respect for one another were mentioned multiple times. Working in the same team regularly was considered important for improved teamwork. Surgeons and scrub nurses commented on the importance of focus on the surgical procedure by all professions. Limiting the number of persons present in the operating room and decreasing the noise level were mentioned to help focus. Many surgeons mentioned intra-operative disruptions, such as coffee breaks for team members as negatively affecting patient safety. It was suggested that everyone in the team should have structured breaks at the same time to avoid distractions. Another suggestion was to have a more flexible system for intra-operative pauses and lunch breaks.

#### Improved management and structure

Comments were made regarding a need for changes at the management level. There were comments on stressful situations due to unsatisfactory staffing levels, such as *‘Inadequate number of operations, due to the staffing of the operating department’.* Some surgeons wanted improved logistics between operations to decrease turnover time. Participants mentioned the importance of adherence to guidelines in order to improve safety. *‘To follow guidelines and evidence based clinical routines’.*

Surgeons, anesthesiologists and nurses asked for more profound knowledge and competence among the operating room personnel. Education, thorough introduction and learning from mistakes were suggested.

## Discussion

In this study we found that operating room team members reported a need for improved teamwork and communication within the team. We found a lack of structure in the usage of the WHO checklist. Based on this a revision of the WHO checklist was devised and implemented. However this revision did not affect the teamwork climate measurements, nor communication and collaboration and we conclude that the intervention did not enhance patient safety. These results may be due to the inability to fully implement the new checklist as observations revealed that adherence to the revised checklist was insufficient. Variability in checklist-compliance is a well-known phenomenon [16, 17].

The hypothesis was that the use of a revised checklist based on suggestions from the focus groups would enhance teamwork and indirectly improve patient safety. Although the open-ended question in SAQ revealed that the participants regarded good communication among operating team members as important there still seemed to be deficiencies. We found that different professions regarded communication and collaboration within their own profession as good, but not to the same extent between professions. It was interesting to find the contradiction that surgeons were the most positive regarding communication and collaboration with other surgeons but given the lowest rating by other team members. Similar results were found by Makary et al who showed that 87% of the surgeons rated communication and collaboration with operating room nurses as good while only 48% of the nurses rated surgeons as good communicators [18]. Lack of adequate communication and collaboration between surgeons and scrub nurses was also reflected in the domain teamwork climate where scrub nurses was the profession which rated teamwork the lowest, a pattern also found in research by Sexton et al. [19].

At SAQ baseline many of the participants commented on the importance of an open climate regardless of profession. Enhanced teamwork was suggested to increase commitment. Previous research has shown associations between leader inclusiveness and team engagement in quality improvement in health care [20]. Adding the item ‘description of the surgical procedure’ to ‘Time out’ was intended to increase the involvement of the team members through greater knowledge of the specific procedure and a feeling of inclusion. Another objective was to improve situational awareness which has been associated with fewer surgical errors [21]. Low levels of shared understanding among professionals in the operating room team may reduce efficient teamwork [22].

At baseline we discovered a need for structure and further education regarding the WHO checklist. There was uncertainty regarding the designated checklist coordinator and what this role included. The nurse assistants found it difficult to initiate ‘Time out’ as their role was insufficiently recognized [23]. To successfully manage the checklist it is important that the checklist coordinator has the support of staff in more senior positions [20, 24]. We instructed the team to acknowledge the nurse assistant as the checklist coordinator and introduced a paper-checklist to be filled out. However, this did not seem to be sufficient, according to observations of how the checklist was used.

We failed to fully implement the revised checklist. Interventions to improve the safety climate require strong commitment and support by the management and initial education and training of employees [25–27]. Previous research has also suggested that success requires the support from at least twenty-five percent of the targeted population [28]. The fact that this intervention was led by a scrub nurse, and that nurse assistants were checklist coordinators can have influenced the results [29]. In the hierarchical hospital system it is important who is the person in charge of the intervention, as senior surgeons are probably more likely to successfully implement a changed routine than nurses are [25, 29]. Including physicians in the tailoring of the checklist facilitates the implementation process [29]. Although we included managers, middle-managers and the operating room team in the intervention, it was probably not enough to have the anticipated effect on teamwork climate. The fact that the anesthesiologists were unwilling to participate in the focus groups may have influenced the outcome. Not participating in the focus groups meant that the anesthesiologists not only missed an opportunity for education regarding the checklist, but also that they did not have input into the revision of the checklist. The anesthesiologist were also the profession with the lowest SAQ response rate, 69% and 40% answered SAQ at baseline and post-intervention respectively. The lack of compliance with the intervention is demonstrated both by the absence of the anesthesiologists in the focus groups and in the post-interventional observations where we found that the team members did not use the checklist as intended. Other limitations to this study were that we did not have a control group. It is also possible that two independent observers, not included in the study design, would have contributed to a higher validity without possible expectancy bias. A strength was that the SAQ was assessed both at baseline and post-intervention enabling intra-individual comparisons. The relatively high compliance indicated that staff found the study important and trusted the design regarding the participants’ anonymity.

## Conclusions

There was no significant change in teamwork climate by the use of the revised WHO checklist. This may be due to insufficient implementation, as a lack of adherence to the WHO checklist was detected as well as lack of participation in the focus group meetings. We found deficiencies in teamwork and communication. Further studies exploring how to improve safety climate are needed.

## Abbreviations

Anesth: Anesthesiologists
CRF: Clinical record form
Nurse an: Nurse anaesthetists
Nurse ass: Nurse assistants
SAQ: Safety attitude questionnaire
SEM: Standard error of the mean change
WHO checklist: World Health Organization surgical safety checklist
WHO: World Health Organization

## References

[1] Weiser TG, Regenbogen SE, Thompson KD (2008). An estimation of the global volume of surgery: a modelling strategy based on available data. Lancet.

[2] Flin RH, O’Connor P, Crichton MD. Safety at the sharp end: a guide to non-technical skills. Burlington, VT; Aldershot, England: Ashgate, 2008.

[3] Stout RJ, Cannon-Bowers JA, Salas E, Milanovich DM (1999). Planning, shared mental models, and coordinated performance: an empirical link is established. Human Factors.

[4] Healey AN, Undre S, Vincent CA (2006). Defining the technical skills of teamwork in surgery. Qual Saf Health Care.

[5] Haynes AB, Weiser TG, Berry WR (2009). A surgical safety checklist to reduce morbidity and mortality in a global population. N Engl J Med.

[6] Rydenfalt C, Ek A, Larsson PA (2014). Safety checklist compliance and a false sense of safety: new directions for research. BMJ.

[7] Sexton JB, Helmreich RL, Neilands TB (2006). The Safety Attitudes Questionnaire: psychometric properties, benchmarking data, and emerging research. BMC Health Serv Res.

[8] Mearns K, Flin R (1999). Assessing the state of organizational safety—culture or climate?. Curr Psychol.

[9] Goras C, Wallentin FY, Nilsson U, Ehrenberg A (2013). Swedish translation and psychometric testing of the safety attitudes questionnaire (operating room version). BMC Health Serv Res.

[10] LÖF LÖF-. Checklista för säker kirurgi. 2009

[11] Makary MA, Sexton JB, Freischlag JA (2006). Patient safety in surgery. Ann Surg.

[12] Profit J, Etchegaray J, Petersen LA (2012). The safety attitudes questionnaire as a tool for benchmarking safety culture in the NICU. Arch Dis Child Fetal Neonatal Ed.

[13] Bowen GA. Naturalistic inquiry and the saturation concept: a research note. Qualitative Research 2008;8(1):137-52. doi:10.1177/1468794107085301[published Online First: Epub Date].

[14] Kitzinger J (1995). Qualitative research. Introducing focus groups. Br Med J.

[15] Graneheim UH, Lundman B (2004). Qualitative content analysis in nursing research: concepts, procedures and measures to achieve trustworthiness. Nurse Educ Today.

[16] de Jager E, McKenna C, Bartlett L, Gunnarsson R, Ho YH (2016). Postoperative adverse events inconsistently improved by the world health organization surgical safety checklist: a systematic literature review of 25 studies. World J Surg.

[17] Gillespie BM, Withers TK, Lavin J, Gardiner T, Marshall AP (2016). Factors that drive team participation in surgical safety checks: a prospective study. Patient Saf Surg.

[18] Makary MA, Sexton JB, Freischlag JA (2006). Operating room teamwork among physicians and nurses: teamwork in the eye of the beholder. J Am Coll Surg.

[19] Sexton JB, Makary MA, Tersigni AR (2006). Teamwork in the operating room: frontline perspectives among hospitals and operating room personnel. Anesthesiology.

[20] Nembhard IM, Edmondson AC (2006). Making it safe: the effects of leader inclusiveness and professional status on psychological safety and improvement efforts in health care teams. J Organ Behav.

[21] Hull L, Arora S, Aggarwal R, Darzi A, Vincent C, Sevdalis N (2012). The impact of nontechnical skills on technical performance in surgery: A systematic review. J Am Coll Surg.

[22] Undre S, Sevdalis N, Healey AN, Darzi A, Vincent CA (2006). Teamwork in the operating theatre: Cohesion or confusion?. J Eval Clin Pract.

[23] World Health Organization. Implementation manual, Surgical Safety Checklist. Secondary Implementation manual, Surgical Safety Checklist 2008. http://www.who.int/patientsafety/safesurgery/tools_resources/SSSL_Manual_finalJun08.pdf?ua=1.

[24] O’Connor P, Reddin C, O’Sullivan M, O’Duffy F, Keogh I (2013). Surgical checklists: the human factor. Patient Saf Surg.

[25] Erichsen AA, Gifford W, Nilsson K (2015). Improving care in surgery — a qualitative study of managers’ experiences of implementing evidence-based practice in the operating room. J Hosp Adm.

[26] Proctor EK, Powell BJ, McMillen JC (2013). Implementation strategies: recommendations for specifying and reporting. Implementation Sci.

[27] Pannick S, Sevdalis N, Athanasiou T. Beyond clinical engagement: a pragmatic model for quality improvement interventions, aligning clinical and managerial priorities. BMJ. 2015. doi: 10.1136/bmjqs-2015-004453 [published Online First: Epub Date]|. 10.1136/bmjqs-2015-004453 PMC5013121 26647411

[28] Rogers EM (2003). Diffusion of innovations.

[29] Gillespie BM, Marshall A (2015). Implementation of safety checklists in surgery: a realist synthesis of evidence. Implementation Sci.

